# Investigation on Combined Inhalation Exposure Scenarios to Biocidal Mixtures: Biocidal and Household Chemical Products in South Korea

**DOI:** 10.3390/toxics9020032

**Published:** 2021-02-04

**Authors:** Sunmi Kim, Myungwon Seo, Minju Na, Jongwoon Kim

**Affiliations:** Chemical Safety Research Center, Korea Research Institute of Chemical Technology, Daejeon 34114, Korea; skim@krict.re.kr (S.K.); mwseo@krict.re.kr (M.S.); mjna@krict.re.kr (M.N.)

**Keywords:** biocidal product, household chemical product, mixture risk assessment, combined inhalation exposure, priority biocide mixture

## Abstract

Global regulations of biocides have been continuously enhanced for protecting human health and the environment from potentially harmful biocidal products. Such regulations consider the combined toxicity caused by mixture components in a biocidal product of which approval and authorization are to be enhanced. Although the combined exposure scenarios of components in mixtures are firstly needed to conduct the mixture risk assessment, systematic combined exposure scenarios are still lacking. In this study, combined inhalation exposure scenarios of biocides in household chemical and biocidal products marketed in South Korea were investigated based on the European Union (EU) and Korean chemical product databases and various data sources integration. The information of 1058 biocidal products and 675 household chemical products that are likely to cause inhalation exposure with two or more biocides was collected, and mixture combination patterns were investigated. Binary mixtures occupied 72% in biocidal products. The most frequently appearing binary mixture was phthalthrin and d-phenothrin. Based on the frequency of use, we suggested a priority list of biocide mixture combinations which need to be firstly evaluated for identifying their combined toxicity for the mixture risk assessment. This study highlights that the derived combined inhalation exposure scenarios can support and facilitate further studies on priority settings for mixture risk assessment and management of potentially inhalable biocides.

## 1. Introduction

Since the unintended fatal lung disease in South Korea was officially reported on August 2011 by Korea Centers for Disease Control and Prevention (KCDC) [1], some humidifier disinfectants containing biocides that can cause the pulmonary fibrosis have triggered 7009 reported victims and 1586 deaths based on applicants for the victim relief, as of November 2020 [2]. This intolerable tragedy substantially showed that the risk assessment covering various uses of chemicals is essential to the chemical product safety management.

Global regulations of biocides have been continuously enhanced for protecting human health and the environment from potentially harmful biocidal products. e.g., European Union (EU) Biocidal Product Regulation (BPR) [3] and Korean Household Chemical Products and Biocides Safety Act (Chemical Product Safety Act, also known as K-BPR) came into force in 2013 and 2019 to effectively improve the safety management of biocidal products [4]. In the Chemical Product Safety Act, the term “Biocides” means the biocidal active substances, which are defined as active chemical molecules controlling the growth of or killing bacteria in a product. The regulation includes two broad categories of chemical products that contain biocides, i.e., biocidal products and household chemical products. In this regulation, the inclusion of two types of chemical products is an attempt to conduct the risk assessment by considering various uses of biocides and to cover the biocides in household chemical products [5]. Thus, after the enforcement of the Chemical Product Safety Act, the management of biocidal chemicals in household chemical products that were previously unregulated became possible. Meanwhile, EU BPR and the Chemical Product Safety Act consider the combined toxicity caused by mixture components in a biocidal product of which approval and authorization are to be enhanced. This is due to the fact that individual chemicals below their no-observed-effect levels may provoke toxicity by toxicological interactions (e.g., additivity, or synergism) in living organisms [6,7,8]. In the risk assessment process of biocidal products, the additive toxicity concept has been frequently employed based on the concentration addition model where there are no evidences of the synergistic toxicity [9]. However, such information on the synergistic toxicity of various biocide mixtures is still very limited, and conducting toxicity tests for all mixture combinations in conventional ways are difficult due to the extremely large number of conceivable mixture combinations. Thus, making prioritization of mixture combinations would be the first step for mixture risk assessment [10].

Although the combined exposure scenarios of components in mixtures are firstly needed to conduct the mixture risk assessment, systematic combined exposure scenarios and corresponding data are still lacking [11,12]. European Chemical Agency (ECHA) provides biocidal active substances, biocidal products, and their suppliers on the ECHA website [13] under the EU BPR. Recently in South Korea, list of all ingredients in some household chemical products were publicly exhibited at EcoLife website [14], and the disclosure was conducted in accordance with the voluntary agreement between the Korean Ministry of Environment and chemical companies. In addition, under the Chemical Product Safety Act, chemical companies manufacturing or importing biocidal products in South Korea notified active substances used in their products to the government by June 2019. These kinds of data can be used not only for building real combined exposure scenarios for the mixture risk assessment, but also for efficient support of mixture toxicity study designs by prioritizing biocide mixtures. In this context, our research interest was placed on combined inhalation exposures to airborne toxicants.

Therefore, the objectives of this study were (i) to investigate potential combined inhalation exposure scenarios of biocides in the household chemical and biocidal products marketed in South Korea, and (ii) to propose a priority list of biocide mixture combinations which are needed to be conducted toxicity testing to identify their combined toxicity. To this end, the active ingredients were listed based on the EU and Korean chemical product databases, and the combined use scenarios and frequently used mixture combinations were investigated to derive priority of toxicity testing for the mixture risk assessment.

## 2. Materials and Methods

### 2.1. Data Collection on Biocidal and Household Chemical Products

Under the Chemical Product Safety Act, the manufacturers or importers should notify the Korea Ministry of Environment of the biocidal ingredients in household and non-household biocidal products. Since biocidal ingredients can be used in ‘biocidal products’ as well as ‘household chemical products’ in South Korea, the following four Korean databases in cooperation with the Korea Ministry of Environment during 2018–2020, and an EU database on authorized biocidal products including active substances were gathered. The EU BPR Database was also used to investigate biocidal products marketed in South Korea by assuming that EU biocidal products could be imported into South Korea. Then all information was intensively analyzed to investigate combinations of biocides:

- Hazard Information and Management System on Biocides by the Korea Ministry of Environment [15] (as of June 2015);
- EcoLife [14], a Household Chemical Products Safety Information System by the Korea Ministry of Environment (1125 products, as of January 2020);
- Biocidal Products Data (unpublished) by the Korea National Institute of Environmental Research (3163 products as of August 2018);
- Biocidal Products Data (unpublished) by the Korea Ministry of Environment (654 products as of February 2020); and
- EU BPR Database by ECHA [16] (1726 authorized products as of January 2019).

### 2.2. Data Curation and Generation of Combined Inhalation Exposure Scenarios

In this study, a biocidal mixture is composed of two or more biocides. Thus, solvents or natural fragrances that have biocidal functions in the products were also considered and involved in investigating the biocidal mixture combinations. Their combinations of biocides in the product are defined as ‘combined exposure scenarios’ of biocides. Based on the collected data, a database of combined exposure scenarios of biocides was generated and curated in Microsoft Excel with the following main data attributes:

- Product information: product name (if any), product category, and type of product (solid (powder), liquid, gel, or spray-type);
- Exposure information: product use, possibility of inhalation exposure, and type of inhalant (particle, aerosol, or volatile); and
- Ingredient information: chemical name, CAS registry number, PubChem ID, EC number, UN number, molecular weight, simplified molecular-input line-entry system (SMILES) information, toxicity, etc.

The product categories were defined in accordance with ‘Chemical Product Safety Act’ as shown in Table 1. For the biocidal products, they were classified into four sub-categories: disinfectants (fungicides, algaecides, and humidifier disinfectants), pest control products (repellents, attractants, insecticides, and rodenticides), preservatives (wood preservatives, and preservative-treated filter) and others (mainly anti-foulants). The household chemical products were classified into six sub-categories: cleaning products (kitchen detergents and removers), laundry products (laundry detergents, bleaches, and fabric conditioners), coatings and adhesives (polishes, anti-fog agents/water repellents, ironing auxiliaries, adhesives and gap fillers), air fresheners (fresheners and deodorants), car care products (car wash, and antifreeze solution), and others (candles, mist eliminators, and snow spray). In a conservative way, it was assumed that the possible inhalation routes of products could be determined based on the types and uses of those products to derive a combined inhalation exposure scenario for biocides.

### 2.3. Selection of Biocides for Analyzing Exposure Scenarios

We obtained the information on 1789 products that had biocidal ingredients. Among them, in total 1733 products (97%) had two or more biocides. By excluding duplicated combinations from them, 768 combined exposure scenarios were finally retained. Then, 1733 biocidal and household chemical products were used as the total data for further data analysis, since the total data reflected the real condition of the market. To analyze and visualize major ingredients frequently used among mixtures, Cytoscape 3.8.2 [17] was used. The degree of frequency between two chemicals (nodes) was calculated by a network analysis tool in the Cytoscape, and the network was generated by the yFiles Organic Layout algorithm.

## 3. Results

### 3.1. Generation of Combined Exposure Scenarios Data

**Figure 1:**
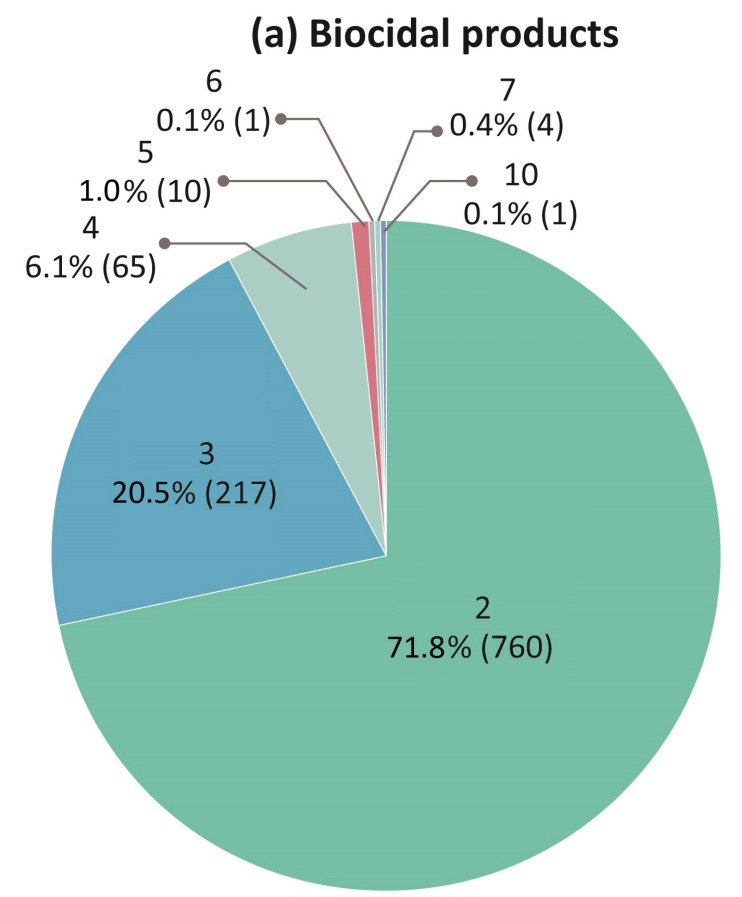

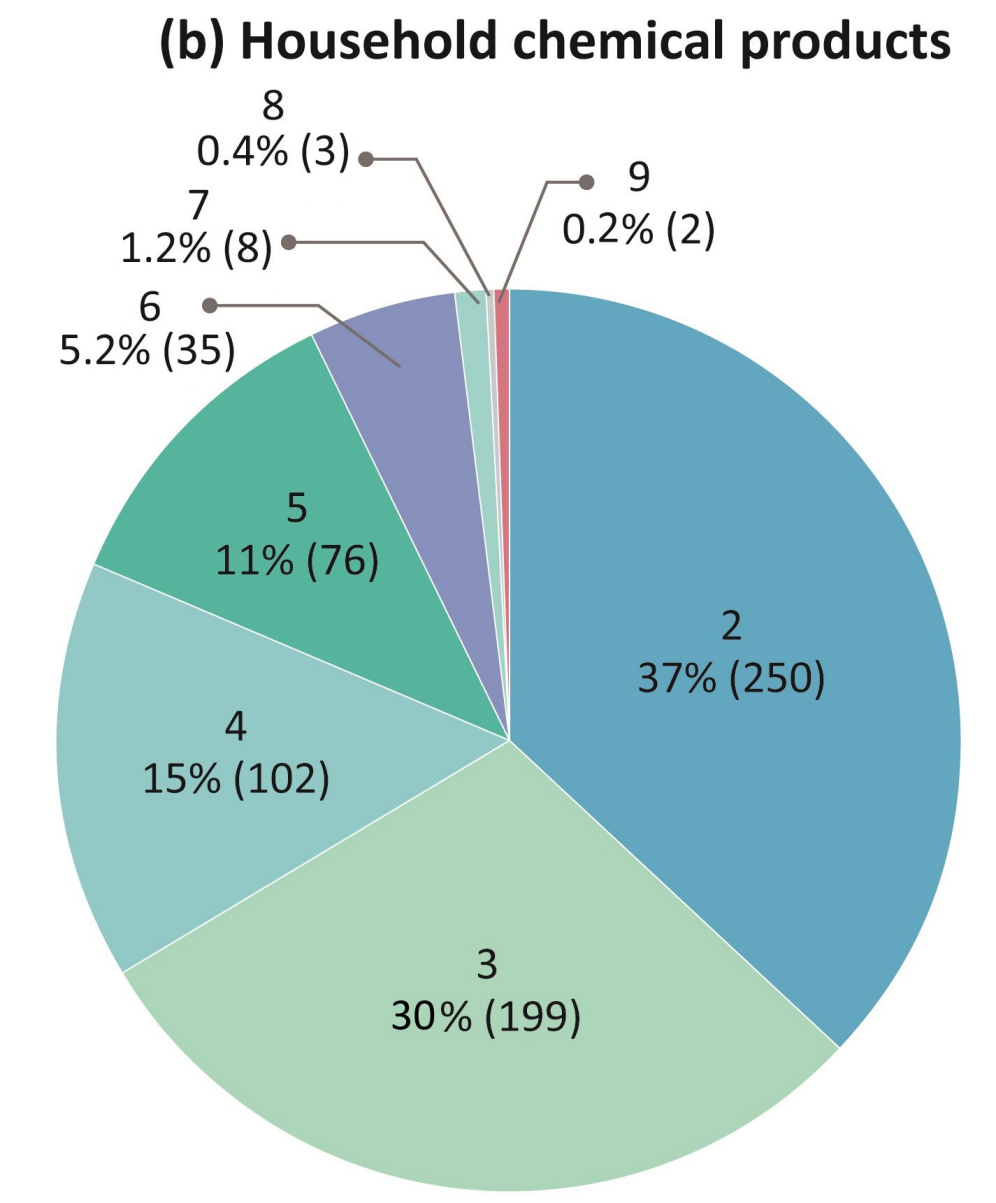
Distribution of the specific product category (**a**) biocidal products; (**b**) household chemical products which have biocidal active substances in the combined inhalation exposure scenario data of biocides in South Korea investigated in this study.

From the 1733 products in the total data, 1058 biocidal products and 675 household chemical products were found and analyzed in this study (Table 2). Based on the total data, 314 biocides were identified as being used in biocidal and household chemical products. Pest control products and air freshener dominated the biocidal products (49.7%), and household chemical products (45.3%), respectively (Figure 1).

**Figure 2:**
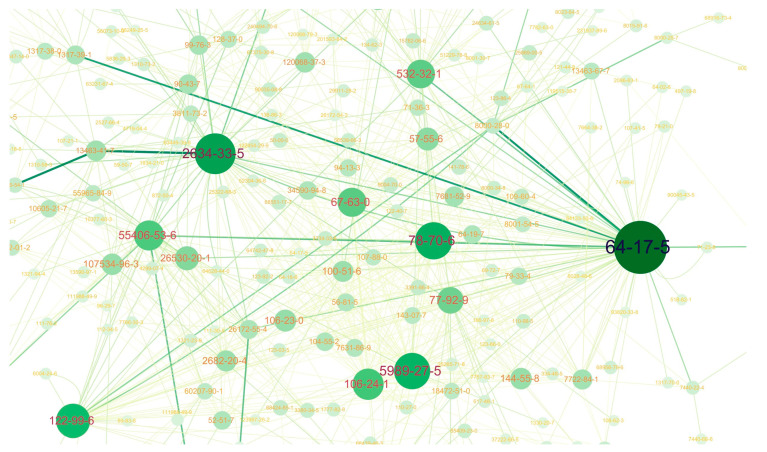
A part of the network visualization of chemical combinations in biocidal and household chemical products. Lines connect ingredients contained in the same product, and color-focused with larger size vertices means more abundantly used chemicals. The numbers in circles represent the CAS registry number of chemicals. Five compounds that have the high node’s degree in this part are shown as follows: 64-17-5 (ethanol), 2634-33-5 (BIT), 78-70-6 (linalool), 5989-27-5 (D-Limonene), and 122-99-6 (2-phenoxyethanol).

Among 314 chemicals, the most common ingredients (its frequency of appearing) were linalool (438), ethanol (335), d-limonene (324), and geraniol (213), which their usages could be natural fragrance or solvent. Next, phthalthrin (174), 1,2-benzisothiazol-3(2H)-one (BIT) (164), sodium bicarbonate (127), 2-methyl-2H-isothiazol-3-one (MIT) (113), dicopper oxide (111), and 5-chloro-2-methyl-2H-isothiazol-3-one (CMIT) (101) were frequently included in more than 100 products (6~10%). 2-phenoxyethanol, and d-phenothrin were used over 90 products (5.2%), and 3-iodo-2-propynyl butylcarbamate (IPBC), sodium benzoate, permethrin, citric acid, hydramethylnon, cypermethrin, 2-pyridinethiol-1-oxide copper salt, (2-methoxymethylethoxy)propanol, 2-propanol, carbendazim, chlorpyrifos were used over 50 products (2.8%). The next common ingredients were used in more than 40 products (2.3%), and they are methylparaben, copper monoxide, fipronil, and prallethrin. All ingredients and their networks were visualized (Supplementary Figure S1) and the main part of the network was presented in Figure 2.

### 3.2. Combined Inhalation Exposure Patterns of Biocides in Biocidal and Household Chemical Products

**Figure 3:**
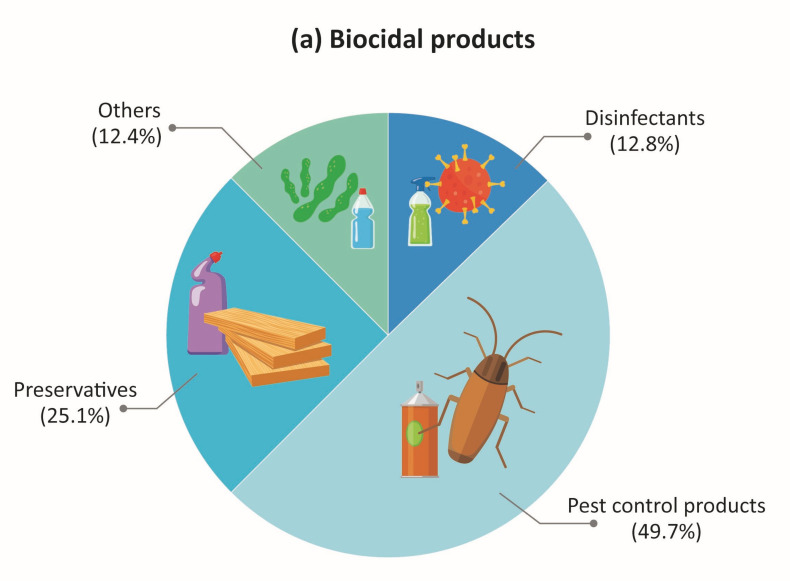

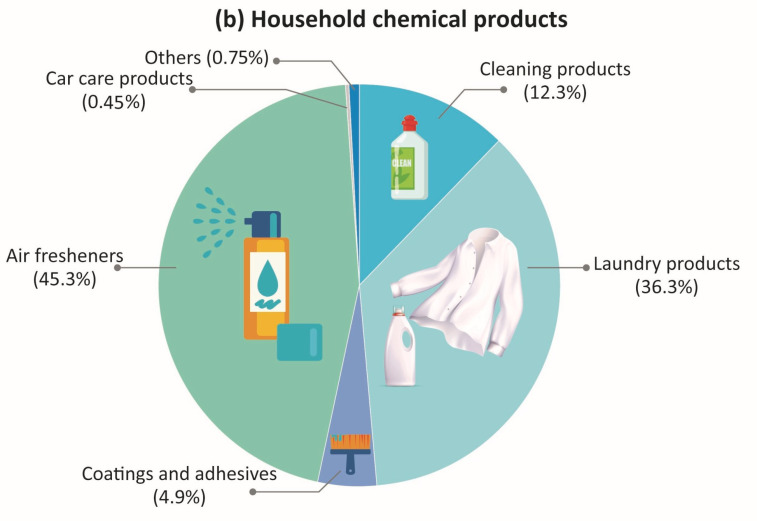
Distribution of the number of biocides in the mixture combinations investigated in the combined inhalation exposure scenarios data of biocides in South Korea (**a**) biocidal products; (**b**) household chemical products.

Investigation on the combined inhalation exposure patterns of biocides in the biocidal and household chemical products was carried out. Results revealed that 93% of the biocidal mixtures were mainly binary and ternary mixtures, as well as 92% of the household chemical mixtures were less than quinaries (Figure 3). In the case of 1058 biocidal mixtures, 760 binary (72%) and 217 ternary (21%) patterns were identified. In the other case of 675 household chemical mixtures, mainly, 250 binary (37%), 199 ternary (29%), and 102 quaternary (15%), and 76 quinary (11%) patterns were analyzed.

In order to suggest the priority list of biocidal mixtures, information on the products, ingredients and exposure scenarios were collected and frequency of occurring mixtures were investigated. To cover as many substances as possible, solvents and natural fragrances reported as having biocidal functions were also included in the total list of biocidal mixtures in this study. However, the mixtures composed of only solvents and natural fragrances (e.g., d-limonene + ethanol, and geraniol + linalool) were excluded during the prioritizing process so that the priority of combined exposures could have at least one or more biocidal active substance.

The most common binary mixtures were examined (Table 3), and the number of occurrence of phthalthrin and d-phenothrin was 71 times. The second most common binary mixture was CMIT and MIT, which are known as major causes of lung disease by the humidifier disinfectants in South Korea [18]. They are currently banned in spray-type household chemical products and fragrances but still can be used in biocidal products or certain type of household chemical products. Other frequently occurring binary mixtures occurred more than 30 times, and they were phthalthrin + permethrin, dicopper oxide + copper pyrithione and IPBC + carbendazim, suggesting combinations with the same ingredients were commonly found in biocidal products.

Among the ternary mixtures, CMIT + MIT + bronopol was the most frequently occurring combination (Table 4) when we exclude mixtures that containing solvent or natural fragrances. Phthalthrin + allethrin + permethrin mixture and copper monoxide + copper pyrithione + dicopper oxide mixture are appearing together in seven products. It is shown that most of the ternary mixtures are included in biocidal products, and only hydramethylnon + propylparaben + methylparben mixture is used in household chemical products.

In this study, 36 senaries, 12 septenaries, three octaries, two nonaries, and one denary were also found. Except for ethanol and natural fragrance, most commonly included chemicals in these mixtures were BIT, copper oxide compounds, and 2-phenoxyethanol. Among the mixtures with four or five biocides, DPGME + D-limonene + ethanol + geraniol + toluene was the most frequently appearing combination (Table 5).

## 4. Discussion

### 4.1. Major Findings and Significance of the Study

Human exposures to biocides might occur primarily via the inhalation or dermal exposure routes [5]. Biocides pose potential risks to the respiratory system, nervous system, skin, eyes, and other specific target organs, especially to vulnerable groups, e.g., pregnant women, unborn fetus, children, or people having serious underlying diseases [19]. Our study focused on the inhalation route, which is an important route of biocidal exposure. Since this study is one of the first investigations of combined inhalation exposure scenarios of biocides chemicals in biocidal and household chemical products marketed in South Korea, we expect that our work highly support the experimental design strategies for the mixture risk assessment. Especially, the risk assessor can effectively prioritize and select frequently used biocides and their combinations based on the combined inhalation exposure scenarios. It is an important issue concerning combined exposures to various chemicals, which could provoke the mixture toxicity due to their cocktail effects although some individual chemicals are managed at safe levels [20]. Independent chemicals in a mixture may adversely affect different organs and these could be evaluated by an individual chemical risk assessment [21]. In cases where similarly acting chemicals that affect the same organs via the same mechanisms, their mixture toxicity might be adequately evaluated by an additive toxicity model [22,23]. Under the EU BPR and Plant Protection Product Regulation [24], the additive toxicity concept has been employed as a default method in the conventional mixture risk assessment unless there are evidences of synergistic toxicity (i.e., greater than the additive toxicity). In cases where mixture components cause the synergistic toxicity, their risk cannot be appropriately evaluated by the conventional mixture risk assessment [6].

Nowadays, due to the COVID-19 pandemic, the use of biocides including disinfectants has increased significantly and accordingly, it is predicted that the exposure of disinfecting chemicals to the general population has also increased [25,26]. Since the use of disinfectants is a necessary for COVD-19 prevention, safety issues became more and more important. In particular, “intentional” use of single and mixture components in household chemical products should be evaluated. Under the EU BPR and the Chemical Product Safety Act in Korea, potential adverse effects of combined exposure to the biocides should be taken into account for safe use of biocidal chemicals. However, this recommendation can be unfeasible in cases where there is limited information on mixture compositions and empirical data on the mixture toxicity [12] since the number of conceivable mixtures is extremely large. Thus, making prioritization strategy of mixture combinations to reduce the number of target mixtures and optimize the experimental design of the mixture toxicity should be the first step for mixture risk assessment and management [10]. In this context, this study provides prioritized biocides and their mixture combinations included in biocidal and household products based on the investigated combined inhalation exposure scenarios.

Several previous studies reported combinations of chemicals in household chemical products in the USA and Germany. Gabb and Blake [27] applied an informatics approach to evaluate combined chemical exposures from the consumer products, using on the on-line market data gathering. Compared to this approach, our data are generated based on nationally notified data excluding confidential data, and likely cover most types of biocidal products. Uter et al. [28,29,30] examined combined exposures of ingredients for each group of fragrance, preservatives, and UV-filters in cosmetic products. In our study, we further comprised a wide range of products containing biocidal ingredients to consider receptor-basis assessment. In South Korea, one recent study used an EcoLife database like our approach and provided a priority list for biocidal chemicals [31]. Choi et al.’s work was of great significance in applying chemical ranking and scoring concept with data collection for commercially available biocidal products. Then by combining the information about hazard and exposure potential, the priority setting was provided. The products covered in their work were only limited to biocidal products, which included 171 ingredients in 989 products in 2018 and their study had no consideration for combined exposure. Data generation to assist in priority setting for biocide mixtures carried out in our study is one of the most important processes in the mixture risk assessment. Our study covered a wider range of products, i.e., household chemical products, and data in more recent years were collected from reliable sources by using data from the Korea Ministry of Environment. If the information from Choi et al.’s study [31] and our study can be further considered together, it will be more feasible to identify mixtures that are urgently needed for mixture toxicity tests.

### 4.2. Limitations and Future Scope

Our study has some limitations: firstly, our data of household chemical products covered 10% (1125) of the total 10,463 household chemical products disclosed on the EcoLife website. This was due to that fact that we only targeted products that are likely to cause inhalation exposure, and also the ingredients information of only 1125 products were voluntarily provided by chemical companies. However, in contrast to the data of household chemical products, our data of the biocidal products covered 100% of the products using registered data in Korea Ministry of Environment, Secondly, the collected data might contain the information on biocidal and household chemical products that were no longer marketed in South Korea since we collected all data continuously during 2018–2020. Thirdly, the biocidal product lists were mainly based on the biocidal products registered in South Korea and authorized in the EU which were assumed to be imported into South Korea. Thus, some biocidal combinations could be excluded or included according to related regulations and regional market situations of the other countries. As of December 2020, 276 chemicals were approved as biocidal active substances in ECHA [32] and possibly most of the substances were included in our total data. Lastly, the priority lists suggested by this study could not consider the quantitative composition of biocides in mixtures. This is due to the confidential information for the industry. However, the ratio of the compositions in the mixture can influence their mixture toxicity even if ingredients are same.

It is expected that our results on the combined inhalation exposure scenarios and their priority will facilitate future studies (i) for conducting the mixture risk assessment based on the priority lists; and (ii) for effectively targeting mixture combinations to generate toxicity datasets and to develop predictive models for screening the potential synergistic toxicity of the mixture components. Based on this study, further studies also need to be carried out for refining priority combination lists by considering chemoinformatic data (e.g., physico-chemical properties, structural information, etc.) and bioinformatic data (e.g, interacting proteins, genes, mechanistic information, etc.).

## 5. Conclusions

This study examined the combined inhalation exposure scenarios of biocidal chemicals placed on the market in Korea, including 1733 products with 314 biocides. In total, 1058 biocidal products and 675 household chemical products were found to contain biocides mixtures. Among the four main product types of the biocidal products, the pest control product was most dominant (49.7% of total products). Among six major product types of the household chemical products, the air freshener was the majority (45.3% of total products). Through this study, we identified frequently used combinations of biocides and the range of number of biocides used in products was 2–10. In both product categories, 93% was binary and ternary mixtures. Considering occurring frequencies of mixtures, product types, and inhalation exposure route simultaneously, we also suggested the priority lists for biocide mixtures. This study highlights that the derived combined exposure scenarios can support and facilitate further studies on priority settings for the mixture risk assessment and management of potentially inhalable biocides in the products.

## Figures and Tables

**Table 1:** Main inhalants having inhalation routes that can be determined by product types and uses.

Broad Category	Sub-Category	Product Type	Inhalant
Biocidal products	Disinfectants	Liquid/gel	Volatile
	Pest control products	Liquid/gel	Volatile
	Preservatives	Solid/powder	Particle
		Liquid/gel	Volatile
		Spray	Aerosol
	Others	Solid/powder	Particle
		Liquid/gel	Volatile
Household chemical products	Cleaning products	Solid/powder	Particle
		Liquid/gel	Volatile
		Spray	Aerosol
		Tissue-type	Volatile
	Laundry products	Solid/powder	Particle
		Liquid/gel	Volatile
		Spray	Aerosol
		Tissue-type	Volatile
	Car care products	Solid/powder	Particle
		Liquid/gel	Volatile
		Spray	Aerosol
		Tissue-type	Volatile
	Air fresheners	Solid/powder	Particle
		Liquid/gel	Volatile
		Spray	Aerosol
	Coatings and adhesives	Solid/powder	Particle
		Liquid/gel	Volatile
		Spray	Aerosol
		Tissue-type	Volatile
	Others	Solid/powder	Particle
		Liquid/gel	Volatile
		Spray	Aerosol

**Table 2:** Prevalence of the biocides in biocidal and household chemical products that had two or more biocides.

Broad Category	Sub-Category	No. of Products (% in Sub-Category)	No. of Biocides
Biocidal products	Disinfectants	135 (12.8%)	100
	Pest control products	526 (49.7%)	111
	Preservatives	266 (25.1%)	111
	Antifouling products	131 (12.4%)	17
	Subtotal	1058 (100%)	286
Household chemical products	Cleaning products	83 (12.3%)	39
	Laundry products	245 (36.3%)	31
	Coatings and adhesives	33 (4.9%)	22
	Air fresheners	306 (45.3%)	37
	Car care products	3 (0.4%)	6
	Others	5 (0.7%)	9
	Subtotal	675 (100%)	68
**Total**		1733	314

**Table 3:** Chemical name and CAS registry number (RN) of Top 15 ^1^ most frequently occurring binary mixtures of biocides from the combined inhalation exposure scenarios database in South Korea.

No.	Chemical Name (1)	CAS RN (1)	Chemical Name (2)	CAS RN (2)	No. of Products Containing This Pair
1	Phthalthrin	7696-12-0	D-phenothrin	26002-80-2	71
2	CMIT	26172-55-4	MIT	2682-20-4	64
3	Phthalthrin	7696-12-0	Permethrin	52645-53-1	38
4	Dicopper oxide	1317-39-1	Copper pyrithione	14915-37-8	37
5	IPBC	55406-53-6	Carbendazim	10605-21-7	35
6	Hydrogen peroxide	7722-84-1	Salicylic acid	69-72-7	28
7	Copper monoxide	1317-38-0	Dicopper oxide	1317-39-1	23
8	Phthalthrin	7696-12-0	Cypermethrin	52315-07-8	16
	Hydramethylnon	67485-29-4	Sodium benzoate	532-32-1	16
9	D-Limonene	5989-27-5	Citronellal	106-23-0	15
10	Dicopper oxide	1317-39-1	DCOIT	64359-81-5	14
11	Imiprothrin	72963-72-5	Cypermethrin	52315-07-8	12
	Fipronil	120068-37-3	Sodium benzoate	532-32-1	12
	Chlorpyrifos	2921-88-2	Methylparaben	99-76-3	12
12	Linalool	78-70-6	Oxydipropanol	25265-71-8	11
	Cyfluthrin	68359-37-5	Cypermethrin	52315-07-8	11
	Imiprothrin	72963-72-5	Cyphenothrin	39515-40-7	11
	Tralopyril	122454-29-9	Zinc pyrithione	13463-41-7	11
13	Silver	7440-22-4	Nano TiO_2_ (Anatase)	1317-70-0	10
14	BIT	2634-33-5	C10-13-iso-Alkanes	68551-17-7	9
	Propylene glycol	57-55-6	Ethyl butylacetyl aminopropionate	52304-36-6	9
	Hydramethylnon	67485-29-4	Methylparaben	99-76-3	9
15	BIT	2634-33-5	2-Propanol	67-63-0	8

^1^ All components in binary mixtures are solvent or natural fragrance (e.g., ethanol, linalool, d-limonene, citric acid and geraniol), those mixtures were removed in this list. Abbreviations. CMIT: 5-chloro-2-methyl-2H-isothiazol-3-one; MIT: 2-methyl-2H-isothiazol-3-one; IPBC: 3-iodo-2-propynyl butylcarbamate; DCOIT: 4,5-dichloro-2-octyl-2H-isothiazol-3-one; BIT: 1,2-benzisothiazol-3(2H)-one.

**Table 4:** Top 10 ^1^ most frequently occurring ternary mixtures of biocides from combined inhalation exposure scenario database in South Korea.

No.	Chemical Name (1)	Chemical Name (2)	Chemical Name (3)	Frequency
1	D-Limonene	Silicon dioxide	Sodium bicarbonate	62
2	D-Limonene	Hydrogen peroxide	Salicylic acid	28
3	D-Limonene	BIT	Ethanol	25
4	D-Limonene	Linalool	Sodium bicarbonate	23
5	D-Limonene	Bentonite	Linalool	14
6	Geraniol	Linalool	Sodium sulphite	11
7	CMIT	MIT	Bronopol	10
8	DPGME	Ethanol	Linalool	8
	2-phenoxyethanol	Ethanol	Linalool	8
9	Phthalthrin	Allethrin	Permethrin	7
	Copper monoxide	Copper pyrithione	Dicopper oxide	7
10	D-Limonene	Citronellal	Ethanol	6
	Phthalthrin	D-phenothrin	Prallethrin	6
	Hydramethylnon	Propylparaben	Methylparaben	6
	Copper monoxide	Dicopper oxide	Zineb	6

^1^ All components are solvent or natural fragrance (e.g., ethanol, linalool, d-limonene, citric acid and geraniol), those mixtures were removed in this list. Abbreviations. BIT: 1,2-benzisothiazol-3(2H)-one; CMIT: 5-chloro-2-methyl-2H-isothiazol-3-one; MIT: 2-methyl-2H-isothiazol-3-one; DPGME: Di(propylene glycol) methyl ether; Zineb: zinc ethylene bisdithiocarbamate.

**Table 5:** Top five most frequently occurring four- or five-way combinations in combined exposure scenario database of biocides in South Korea.

No.	Chemical Name (1)	Chemical Name (2)	Chemical Name (3)	Chemical Name (4)	Chemical Name (5)	Frequency
1	DPGME	D-Limonene	Ethanol	Geraniol	Toluene	30
2	Zineb	Copper monoxide	Dicopper oxide	Copper	-	9
3	D-Limonene	BIT	Ethanol	Linalool	-	8
	D-Limonene	Ethanol	Linalool	2-Propanol	Silicon dioxide	8
4	Copper monoxide	Dicopper oxide	Copper pyrithione	DCOIT	Copper	6
5	D-Limonene	2-phenoxyethanol	Citronellal	Ethanol	-	5
	Ethanol	Geraniol	Linalool	Oxydipropanol	-	5
	BIT	2-phenoxyethanol	L-(+)-lactic acid	Lauric acid	-	5
	D-Limonene	BIT	Ethanol	Silicon dioxide	Sodium bicarbonate	5

Abbreviations. DPGME: Di(propylene glycol) methyl ether; BIT: 1,2-benzisothiazol-3(2H)-one; DCOIT: 4,5-dichloro-2-octyl-2H-isothiazol-3-one; Zineb: zinc ethylene bisdithiocarbamate.

## Data Availability

Restrictions apply to the availability of these data. A part of essential data on biocidal products was obtained from the Korea Ministry of Environment for research purpose only. Thus, those data can be accessible with the permission of the Korea Ministry of Environment. Data on household chemical products in this study are publically available at Ecolife website (https://ecolife.me.go.kr, in Korean).

## Supplementary Materials

The following are available online at https://www.mdpi.com/2305-6304/9/2/32/s1, Figure S1: High-resolution image of the network visualization of chemical combinations in biocidal and household chemical products.
