# Supplementary material for: Investigation on Combined Inhalation Exposure Scenarios to Biocidal Mixtures: Biocidal and Household Chemical Products in South Korea
Source: Toxics. 2021 Feb 4;9(2):32. doi: 10.3390/toxics9020032 (PMC7913984; doi:10.3390/toxics9020032)
Supplement: Supplementary file 1 [file toxics-09-00032-s001.pdf]

Supplementary Materials: Investigation on Combined Inhalation Exposure Scenarios to Biocidal Mixtures: Biocidal and Household Chemical Products in South Korea

Sunmi Kim, Myungwon Seo, Minju Na and Jongwoon Kim

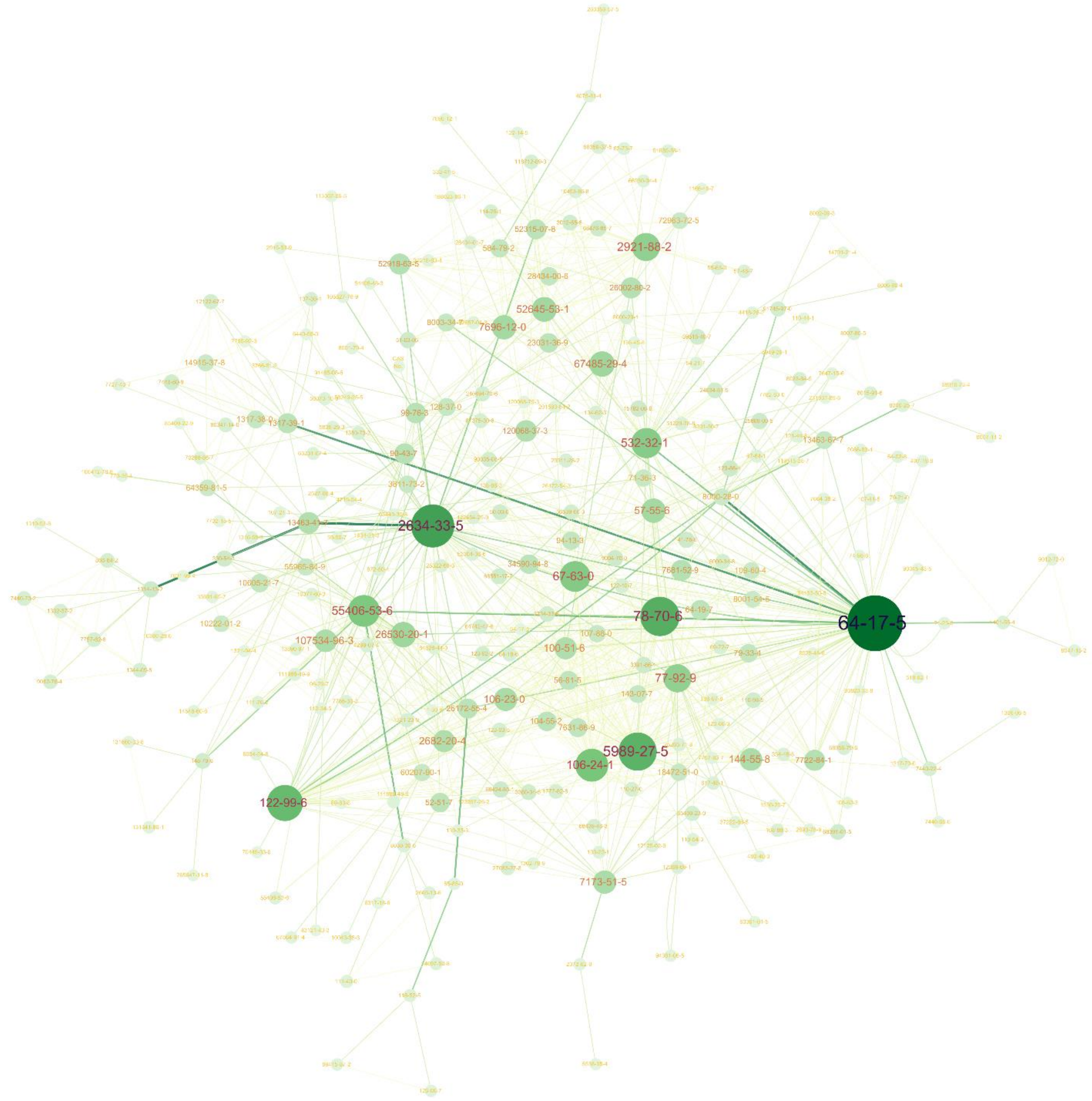

Figure S1. High-resolution image of the network visualization of chemical combinations in biocidal and household chemical products. Ingredients contained in the same product were connected by lines, and the numbers in circles represent the CAS registry numbers. In the network, the node's degree is the number of the link connected with neighbors of nodes, and nodes with a value of a high degree are identified as the important nodes. We visualized the size and color of the nodes based on the degree of the nodes, and the larger size and darker color nodes can be interpreted more important nodes relatively.
